# Using Floating Car Data to Analyse the Effects of ITS Measures and Eco-Driving

**DOI:** 10.3390/s141121358

**Published:** 2014-11-11

**Authors:** Alvaro Garcia-Castro, Andres Monzon

**Affiliations:** 1 Transport Research Centre (TRANS*y*T), Escuela de Ingenieros de Caminos, Canales y Puertos, Universidad Politécnica de Madrid, Ciudad Universitaria, Madrid 28040, Spain; 2 Transport- Civil Eng. Department, Escuela de Ingenieros de Caminos, Canales y Puertos, Universidad Politécnica de Madrid, Ciudad Universitaria, Madrid 28040, Spain; E-Mail: andres.monzon@upm.es

**Keywords:** CO_2_ emissions, speed profiles, traffic simulation, vehicle tracking, smartphones, variable speed limits, section speed control, cruise control, eco-driving

## Abstract

The road transportation sector is responsible for around 25% of total man-made CO_2_ emissions worldwide. Considerable efforts are therefore underway to reduce these emissions using several approaches, including improved vehicle technologies, traffic management and changing driving behaviour. Detailed traffic and emissions models are used extensively to assess the potential effects of these measures. However, if the input and calibration data are not sufficiently detailed there is an inherent risk that the results may be inaccurate. This article presents the use of Floating Car Data to derive useful speed and acceleration values in the process of traffic model calibration as a means of ensuring more accurate results when simulating the effects of particular measures. The data acquired includes instantaneous GPS coordinates to track and select the itineraries, and speed and engine performance extracted directly from the on-board diagnostics system. Once the data is processed, the variations in several calibration parameters can be analyzed by comparing the base case model with the measure application scenarios. Depending on the measure, the results show changes of up to 6.4% in maximum speed values, and reductions of nearly 15% in acceleration and braking levels, especially when eco-driving is applied.

## 1. Introduction

### 1.1. Transportation Sector Emissions

Greenhouse gas (GHG) emissions and overconsumption of energy resources pose a global problem, which concerns both their causes and consequences [1]. The transportation sector is one of the largest emitters despite the advances in the field of engine technology. According to statistics provided by the European Environment Agency [2], GHG emissions from the road transportation sector have started to decline, but still account for about 93% of the emissions attributable to the transportation sector, and approximately 20.4% of total emissions. This value is slightly higher than reported for other developed countries such as Japan [3]. In the US, the contribution of road transportation to total GHG emissions is even higher, reaching almost 22% [4]. The transportation sector accounted for 26% of global energy consumption in 2010, and transportation energy use is expected to increase by 1.1% every year from 2010 to 2040 according to the International Energy Outlook 2013 Reference case [5].

In addition to the global problem of GHG emissions, another major public concern is air quality in urban areas. According to the European Environment Agency, during the period 1997–2008 up to 62% of the European population living in cities (70% of the total population) may have been exposed to concentrations of particulate matter, ozone or nitrogen dioxide above EU-established air quality limits. Transportation is widely recognized to be a significant source of air pollution, especially in metropolitan areas where urban transportation accounts for 70% of pollutants [6].

In this context, much of the effort dedicated to reducing energy consumption and emissions has focused on the road transportation sector. The European Commission [7] proposes an integrated policy to tackle the problem from different approaches; these include particularly demand management, a shift to cleaner modes, improving vehicle technologies, traffic management, and the use of information and communication technologies (ICT). ICT applied to transportation (Intelligent Transportation Systems, or ITS) is a broad field that has the potential for producing positive effects on efficiency, safety, comfort and the environment [8].

### 1.2. Modelling and Simulation of Road Traffic Emissions

The implementation of certain ITS on roads and in vehicles may entail major investments, and it is therefore crucial to predict their potential impacts in advance. *Ex-ante* modelling is a very cost-effective tool which can be useful in selecting the best design, thereby avoiding unnecessary on-site tests. The simulation process in the case of emissions assessment requires a combination of traffic and emission models, which is not a straightforward process.

Fuel consumption and emissions models can be classified based on their level of aggregation. According to Treiber and Kesting [9], these range from aggregated models normally used for national or regional emissions inventories, to the most microscopic modal emission models.

From a macroscopic point of view it can be assumed that demand, fleet composition and average speed determine the level of emissions [10]. However, a detailed look at each vehicle reveals that acceleration and deceleration processes and engine performance also play a critical role [11].

In this framework, the ICT-Emissions research project [12] aims to find a methodology to produce a detailed simulation of the effects of a number of ITS measures on emissions. Evidently each of the ICT measures considered affects not only the traffic flow, but also the vehicle dynamics and driving behaviour, and thus has a considerable influence on fuel consumption and emissions. The impact assessment must therefore be based on detailed models such as speed profile models, as shown in this research. Minor changes in acceleration or speed levels may cause considerable variations in emission rates, which makes the need for accuracy paramount when building and calibrating micro traffic models.

Micro traffic models are traditionally calibrated with aggregated traffic data such as average speed and traffic flow [13], which may not reflect changes in individual driving behaviour, and thus in fuel consumption and emissions. Calibration processes including real vehicle trajectories have become more common in recent years, although few methodologies are available in the scientific literature. Worth noting is the work of Treiber and Kesting, who present a systematic approach to the whole calibration process, which includes the use of Floating Car Data (FCD) and Extended Floating Car Data (xFCD) [14]. A number of other authors investigate the validity of car-following models using FCD; Punzo and Simoneli [15] compared different car-following models with real data obtained from floating cars driving in platoons, similar to the methodology used in [16]. Along the same lines, Kesting and Treiber [17] focused their research on the calibration of car-following models by means of floating vehicles equipped with front radar.

All these studies provide interesting results on the validity of these models under general driving conditions and behaviours. In the present article, FCD is used to analyse the impact of certain ITS measures on driving patterns.

This paper is organized as follows: after explaining the research objectives and methodology (Section 2), Section 3 outlines the tracking and data collection campaign, the measures tested and the data processing. Section 4 briefly describes the traffic micro simulation tools and their calibration parameters. The results of the data analysis and discussion are presented in Section 5. The article ends with some conclusions and final remarks in Section 6.

### 2. Objectives and Methodology

As mentioned in the previous section, the accuracy of the speed and acceleration values is critical when calculating road traffic emissions at the micro scale. The level of accuracy is even more important in the simulation process of certain ITS measures, as it could affect some of the model parameters that would otherwise be considered fixed.

This research therefore aims to analyse the changes in some key variables of real speed profiles as a result of applying certain measures to traffic, vehicles and drivers. The methodology is based on tracking equipped vehicles, and the results serve as a reference for adjusting the simulation parameters in other application areas with similar characteristics.

In summary, the methodology comprises the following steps, which will be explained in detail in the sections below:

- Selection and description of application sections and measures to be tested
- Tracking and data collection methodology
- Trip performance and Floating Car Data acquisition
- Data processing
- Calculation of the speed profile variables applicable in the calibration process of the traffic micro simulator
- Comparison of results between the base case and the different application scenarios.

## 3. Data Collection and Processing

### 3.1. The Case Study of Madrid

Madrid is a city of about 3.5 million inhabitants, with 6 million in its greater metropolitan area. The city is surrounded by three ring motorways: M-30, M-40 and M-50, this last incomplete. The renovation works on the inner M-30 ring, also known as Calle30, were completed in 2007 with the aim of improving traffic performance, reducing pollutant levels and creating new public spaces [18]. Now the ring is a 3 + 3 lane (depending on the sections) urban motorway which extends for 38 km, including 12 km of tunnels. The road is equipped with the most advanced ICT-based equipment such as variable message signs, traffic sensors and vision cameras, which allows stakeholders to collect real-time information. All this equipment is managed from a 24-h control centre.

The speed limit is 90 km/h in surface sections, except for about 3 km of the north section which is a signalized urban avenue where the speed limit is 50 km/h. The speed limit in the tunnel sections is 70 km/h. The average daily traffic ranges from 65,000 to 265,000 vehicles/day, depending on the section, which implies traffic control measures, as well as safety problems. We decided to focus the Madrid case study on ICT measures applicable to motorway sections and adjacent areas with the aim of improving traffic performance, safety and emission levels.

### 3.2. Review of Tested Measures in the Case Study of Madrid

Four different measures were selected for testing in the Madrid case study. Two measures affect only individual vehicles: eco-driving affects driving behaviour, while cruise control is related to vehicle technology. The other two measures involve traffic control and management and depend on the available ITS installations in the road network. We selected Section Speed Control and Variable Speed Limits on account of their availability for experimentation. The following paragraphs contain the definition and a short review of other experiences for each of the measures, while the results are shown in Section 5.

- Section Speed Control
  Section Speed Control (point-to-point speed control or average speed control) is a measure designed primarily to improve road safety, although it may also have effects on traffic performance and emission levels. The number plates of each vehicle entering a section (usually one without intersections) are read and stored at the beginning and end of the section. By measuring the time elapsed, average speed is calculated for each vehicle, and drivers exceeding the legal speed limit are sanctioned in accordance with applicable regulations.
  Apart from road safety, these systems also have an impact on emissions as they prevent speeding, and make traffic flow more uniform. Soole *et al.* [19] collected studies showing that CO_2_ emissions are reduced by 11% to 29% on certain motorway sections in the United Kingdom, with a considerable but variable reduction in vehicles travelling at over the speed limit; while more modest fuel savings of about 5% are estimated in the Naples area (Italy) [20].
- Variable Speed Limits
  Variable speed limits (in this case also called dynamic speed limits) use real-time data (intensity, speed, environmental conditions and so on) to dynamically change the speed limit, adapting it to the circumstances of the road and its environment. Through a control centre and variable message signs, drivers are informed of both recommended and mandatory speed limits. This system is often used on highways with high traffic levels and/or highly variable weather conditions. Under various scenarios based on simulation, Zegeye *et al.* [21] showed that potential fuel savings and CO_2_ emissions range between 3% and 20%, while savings in travel time and increases in throughput are summarised in [22].
- Cruise Control
  All driver assistance systems that influence speed control (or acceleration and deceleration processes) have effects on fuel consumption and emissions. Cruise control is a driver-activated control system that maintains a constant vehicle speed, avoiding unnecessary speed changes which cause additional fuel consumption. This measure is applicable only on high-capacity roads when the traffic intensity is relatively low. According to the review of Klunder *et al.* [23], the potential reduction in CO_2_ emissions varies between 5% and 10%.
- Eco-Driving
  Eco-driving is a driving style aimed at lowering fuel consumption and producing a proportional decrease in CO_2_ emissions. Eco-driving characteristics are generally well defined and easily typified and, according to Barkenbus [24], involve shifting up between 1500 and 2500 rpm (depending on engine technology), maintaining uniform speed as much as possible, anticipating traffic flow and traffic lights, reducing acceleration and deceleration, and avoiding engine idling.
  Driver learning and use of these techniques can be enhanced with on-board information systems such as FIAT eco:Drive [25] which indicate the ideal time to shift gear, or offer recommendations (feedback) after analysing the speed profiles. Eco-driving techniques are most useful in urban areas with high traffic densities and/or traffic signals, where fuel savings can be achieved without reducing average speeds or increasing average travel times.
  A number of studies place individual fuel savings at between 5% and 10%, and in some cases as much as 20% [25–27]. The variability of these data depends largely on the characteristics of the traffic and roads in each case study. The driver's ability to learn these techniques and the vehicle's sensitivity to minor changes in driving style may also have a significant influence.

### 3.3. Vehicle Tracking for Data Collection

**Figure 1.**
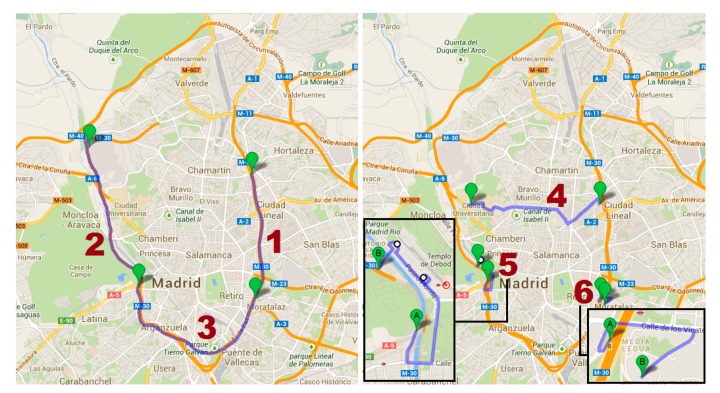
Monitored itineraries in the data collection campaign in Madrid.

The data collection campaign took place in March and April 2013, in coordination with the Department of Traffic Technologies of Madrid. The main objective was to obtain vehicle-related data on various itineraries of the M30 ring motorway (itineraries 1, 2, and 3) and some adjacent urban streets (itineraries 4, 5, and 6) (Figure 1).

As in the case of Madrid, many motorways and urban roads are equipped with cameras and induction loops which record traffic flow parameters such as intensity and average speed at certain points. This data may be enough to gain a picture of the general traffic conditions at a macro level, but it lacks the necessary level of detail to determine patterns at a micro level.

The Floating Car Data method is used to determine traffic and vehicle parameters based on information collected from on-board devices. Floating cars work as moving sensors and do not require significant investment in instrumentation installed on the roadway. This method provides information about individual travel and driving behaviour, simultaneously complementing the information from other fixed on-road sensors.

For many applications, the speed and GPS information recorded by smartphones may be enough. However, the vehicles participating in this campaign also provided information extracted from the on-board diagnostics (OBD) system, as engine parameters needed to be recorded for other project requirements.

**Figure 2.**
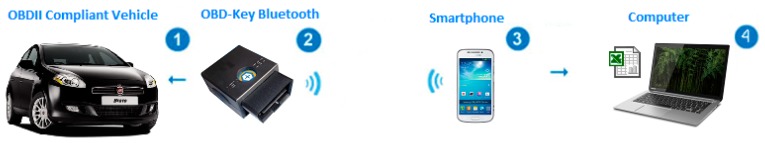
Data collection process.

The methodology included the installation of an OBD key to extract data from each vehicle's OBD system. This was used to record instantaneous speed, acceleration, fuel consumption (l/h down to a precision level of five significant digits) and revolutions per minute (rpm). This device is easily installed by the user in the vehicle diagnostic port, and sends the data to a mobile phone via Bluetooth with a frequency of 1 Hz (Figure 2).

Simultaneously, the mobile phone also recorded GPS coordinates every second. Finally, the data was extracted in .csv format using an application developed by CRF FIAT, one of the partners in the ICT-Emissions project. GPS coordinates were used to select itineraries and split composite trips into urban and motorway sections. There were nine drivers involved and the total number of recorded trips was approximately 3000, which translates into around 12,000 km travelled. The distribution of the total trips is summarised in the following table (Table 1).

Approximately 11% of the trips were initially rejected due to reception failure or inconsistent values. Once the selected itineraries were filtered, the remaining 1562 records were processed in order to obtain the values for 50 variables for each trip, calculated according to traffic and emissions literature [28,29]. Based on the instant speed values recorded, variables can be calculated for each trip, including maximum speed, mean speed, maximum acceleration, maximum deceleration, number of stops and so on, and other statistically derived values such as standard deviation, 95th percentile, among others. Idling time, average rpm, coasting time and other engine variables were also calculated as they are useful for further research into fuel consumption and emissions.

Some of these variables can be considered direct inputs for traffic micro models, while others may be important during the calibration process, especially when assessing the effects of certain measures on emission levels. After the analysis of micro traffic models, Section 4.2 discusses the four selected variables in detail and their correspondence with traffic model parameters.

## 4. Traffic Models and Representative Parameters

### 4.1. Traffic Simulation Models

Traffic microscopic models describe the movements of individual vehicles as the result of individual disaggregate choices and interactions with other vehicles and with the road environment. Software tools simulate the driving behaviour of every single vehicle in every single time step based on different models: car following, lane change, route decision, speed and acceleration decisions, and so on.

Traffic microscopic models require a large amount of detailed input. Some of the parameters of these models can be adjusted by the user as an initial model input or during the calibration process. This allows more accurate simulations in both the base case and in each specific scenario.

Over the last decades, improvements in computer processing power have made possible the development of traffic microscopic models. At present, there are numerous software tools available using different approaches, both open source simulation packages like SUMO and commercial tools like AIMSUM, VISSIM, PARAMICS and TRANSMODELER. Although the approaches and behavioural models may differ, the principle of these tools is quite similar, as are the inputs and calibration parameters. The analysis in this article is based on two of the most widespread software tools: VISSIM and AIMSUM.

VISSIM [30] is a microscopic, behaviour-based multi-purpose traffic simulation program. It is based on Wiedemann's [31] car-following model with time steps as low as 1/10 second and a calibrated lane-changing model for urban and motorway/freeway traffic. Different driving behaviours (*i.e.*, defensive/aggressive) are also supported in the model by adjusting the car-following and lane-change model parameters.

The AIMSUN [32] microscopic simulator is one of the most widespread traffic micro-simulation software tools. It uses traffic flows or Origin-Destination matrices, and allows an easy calibration process through the use of local parameters. It is based on Gipps' car-following model [33].

### 4.2. Parameters for Model Calibration Extracted from Real Speed Profiles

We carried out a review of all the dynamics and behaviour-related parameters that can be adjusted by VISSIM and AIMSUM users, and identified desired speed, acceleration and deceleration with their corresponding variables, extracted from a speed profile. In the case of driving behaviour, the model user is able to adjust parameters such as reaction time, safety distance and aggressiveness which are not directly measurable from a speed profile, although they have a major influence on driving patterns. As the final aim is to accurately estimate emissions and fuel consumption, another variable that correlates well with emissions [22] was selected as a reference to calibrate car-following and lane-changing models inside VISSIM and AIMSUM. The following paragraphs describe the variables selected and their correspondence with the parameters in these two models.

- 95th Percentile of Instantaneous Recorded Speed
  The maximum desired speed is the speed the driver intends to maintain if traffic and road conditions allow. It is related to the speed limit, but it cannot be directly inferred from these values.
  In our case, the maximum desired speed might be taken as the maximum speed recorded in each free-flow trip. However, the selected value corresponds to the 95th percentile of instantaneous recorded speed (VP95), so any minor distractions the driver might have are isolated.
  Desired speed is an essential direct input in the traffic micro simulator. VISSIM presents a desired speed distribution for each vehicle class that can be changed manually by the user, while AIMSUM shows the normal distribution of desired speed in which the user can adjust the mean, deviation, and maximum and minimum values. Following this distribution approach, the mean, standard deviation, and maximum and minimum values have been calculated for each measure and all recorded trips.
- Maximum Recorded Acceleration
  As with the maximum desired speed, the maximum desired acceleration is below the physical maximum acceleration the vehicle is able to achieve if the driver steps on the pedal. It is therefore directly related with the aggressiveness of the driver. It is calculated as the maximum value of the positive recorded acceleration.
  In many of the micro simulation tools reviewed, maximum acceleration is taken as the maximum physical capacity of each vehicle type. In this case, only VISSIM is able to distinguish between maximum acceleration and desired acceleration. AIMSUM only allows the physical maximum acceleration of the vehicle to be changed directly, but not the acceleration desired by the driver.
- Maximum Recorded Deceleration
  Maximum recorded deceleration is calculated as the maximum braking acceleration recorded for each trip. Both VISSIM and AIMSUM allow the desired or normal deceleration distribution to be adjusted in the same way as the desired speed distribution.
- Positive Accumulated Acceleration per Kilometre
  Following the definition found in Garcia-Castro and Monzon [22], this variable reflects the driver's tendency to maintain a constant speed, and shows good correlation with fuel consumption and emissions. The lower the value of this variable, the more constant the speed. This is not a variable that can be used as direct input in any of the models, as it also depends on traffic conditions. However, its variation can be useful to indicate whether the vehicle is travelling more homogeneously as a result of the application of a particular measure. It can be indirectly used as a value for the calibration of different car-following model parameters such as headway or gap acceptance. Table 2 shows a summary of the variables analysed in the following section.

## 5. Effects of ITS Measures and Eco-Driving on Vehicle Trajectories

This section provides a detailed analysis of the FCD recorded. For each of the measures tested, the values of the selected variables (Table 2) were calculated before (base case) and after the implementation of the measure. These results and the variation percentage are shown in Tables 3, 4, 5, 6 and 7, which also includes the results of fuel consumption (l/100 km). Significance values are also reported based on the results of one-way ANOVA tests for each variable (VP95, Amax, Bmax, PAA-km and Fuel Consumption).

### 5.1. Section Speed Control

The section analysed corresponds to a multilane ring motorway, varying from 3 to 4 lanes and 3 or 4 adjacent service lanes. The speed limit is 90 km/h and the total length is 5.8 km. A total of 488 trips were recorded in this itinerary, 262 of which corresponded to the base case and 226 to the section speed control activated scenario. The results are shown in Table 3.

It is worth noting that enforcement produces a 23% reduction in the standard deviation values of the 95th percentile of instantaneous speed, representing a more homogeneous distribution of speed. The higher maximum braking values when the enforcement is activated can be explained by drivers' tendency to brake when the enforcement section begins.

### 5.2. Variable Speed Limits

The section analysed corresponds to a 3-lane ring motorway with a posted speed limit of 90 km/h, except for the last 300 m section which has a posted limit of 70 km/h. As a function of the speed recorded downstream, the variable message sign displays recommended speed limits of 80, 70, 60, 50 or 40 km/h. It is important to note that in the Madrid case study, the speed limits displayed in the variable message signs along the motorway are only a recommendation. Drivers react lowering their speed although the results do not show relevant differences among the recommended speed displayed; only between not activated (base case) and activated. The length of the section under analysis is 6.7 km. Of a total of 336 trips, 177 were recorded with the Variable Speed Limits deactivated, while the system was operative in the other 159. The results obtained are shown in Table 4.

In the case of variable speed limits, no influence is observed on mean desired speed, while the extreme values tend to be closer to the mean, reducing the standard deviation by 23.8%. However, acceleration and braking values are higher, probably as a result of driver reactions to variable message signs.

### 5.3. Cruise Control

Cruise Control is measure that is applicable only to individual vehicles on certain roads and under specific traffic conditions, and particularly only on high-capacity roads when traffic intensity is relatively low. The section under study corresponds to a multilane ring motorway, with 3 or 4 lanes and a normal speed limit of 90 km/h and with a tunnel section with a strictly enforced limit of 70 km/h. The length of the section analysed is 21.3 km. Almost 277 km were driven with the system deactivated, and 298 km when the system was activated by the driver. The results obtained are shown in Table 5.

Cruise control enables the driver to control speeding more effectively, and reduces the maximum speed by around 1.5 km/h. Maximum braking and acceleration values are reduced, although the largest variation in this case is in accumulated acceleration, as the system avoids the minor oscillations that occur under normal driving behaviour conditions.

### 5.4. Eco-Driving

Eco-driving behaviour was tested on different urban and motorway sections, so the results are aggregated by urban or motorway itineraries. Table 6 below presents aggregate values for motorway itineraries in which 212 trips correspond to normal behaviour and 168 were driven following eco-driving recommendations.

The results show that eco-driving in metropolitan motorways has almost no influence on average maximum speed, although the standard deviation is reduced considerably. Average maximum acceleration and deceleration values are considerably reduced, while at the same time the speed profile is much more homogeneous, as shown by the reduction in accumulated acceleration.

For urban itineraries, almost the same kilometres are recorded with normal driving behaviour and eco-driving behaviour from a total of 776 km. The results are shown in Table 7. In the case of urban itineraries, the mean of the 95th percentile of instantaneous speed shows a clear reduction of 6.4%, as do the mean values of acceleration and braking. Speed profiles are also considerably more homogeneous according to the Positive Accumulated Acceleration indicator.

## 6. Conclusions

Traffic modelling is one of the most widely-used tools for forecasting the effects of measures affecting road transportation. However, the calibration process and the accuracy and reliability of the results are still questioned within the research community.

These issues can be even further compounded when evaluating the effects of certain measures on emission levels, since emission models are highly sensitive to minor changes in speed or acceleration rates. The results presented in this article provide a basis for the simulation of the selected measures in other similar areas where specific FCD is not available.

However, it is important to highlight that due to the local nature of the microscopic model, the input values depend to a large extent on the particularities of each case study. For instance, fleet composition and driver behaviour vary from one area to another, while the desired speed depends largely on the characteristics of the infrastructure and speed limit compliance. Thus the main aim of this article is not to provide direct input values, but to offer a percentage of change as an order of magnitude when a similar road or network is affected by any of the ICT measures analysed.

Although this article tests a limited number of measures, it describes a methodology that can be used to collect and analyse FCD to obtain reference values for traffic models, and be applied to any other ITS measure in any other application area. The advances in smartphone technologies and their increasingly generalised use have made it easier to track vehicle trajectories, thus offering more opportunities for applications relating to the accuracy of traffic and emission models.

## Figures and Tables

**Table 1.** Areas of implementation according to Figure 1, number of valid trips and average length per tested ICT measure.

**Areas**	**ICT Measure**	**Type of Section**	**Number of Trips**	**Average Length (km)**
**1**	Section speed control	Motorway	488	5.8
**2**	Variable speed limits	Motorway	336	6.7
**1 + 2 + 3**	Cruise control	Motorway	27	21.3
**1**	Eco-driving	Motorway	218	5.8
**2**	Eco-driving	Motorway	162	6.7
**4**	Eco-driving	Urban	58	7.0
**5**	Eco-driving	Urban	162	2.8
**6**	Eco-driving	Urban	111	1.2

**Table 2.** Variables extracted from the recorded driving profiles and their correspondence with VISSIM and AIMSUM parameters.

**Variable**	**Abbreviation**	**Units**	**Corresponding Calibration Parameter in VISSIM**	**Corresponding Calibration Parameter in AIMSUM**
95th percentile of instantaneous recorded speed (Mean)	VP95_mean	km/h	Desired speed distribution (manual adjust)	Mean desired speed
95th percentile of instantaneous recorded speed (Deviation)	VP95_Sd	km/h	Desired speed distribution (manual adjust)	Standard deviation Desired Speed
95th percentile of instantaneous recorded speed (Maximum)	VP95_max	km/h	Desired speed distribution (manual adjust)	Maximum desired speed
95th percentile of instantaneous recorded speed (Minimum)	VP95_min	km/h	Desired speed distribution (manual adjust)	Minimum desired speed
Maximum recorded acceleration (Mean)	Amax_mean	m/s^2^	Desired acceleration distribution (manual adjust)	Mean desired acceleration
Maximum recorded acceleration (Deviation)	Amax_Sd	m/s^2^	Desired acceleration distribution (manual adjust)	Standard deviation desired acceleration
Maximum recorded acceleration (Maximum)	Amax_max	m/s^2^	Desired acceleration distribution (manual adjust)	Maximum desired acceleration
Maximum recorded acceleration (Minimum)	Amax_min	m/s^2^	Desired acceleration distribution (manual adjust)	Minimum desired acceleration
Maximum recorded deceleration (Mean)	Bmax_mean	m/s^2^	Desired deceleration distribution (manual adjust)	Mean desired deceleration
Maximum recorded deceleration (Deviation)	Bmax_Sd	m/s^2^	Desired deceleration distribution (manual adjust)	Standard deviation desired deceleration
Maximum recorded deceleration (Maximum)	Bmax_max	m/s^2^	Desired deceleration distribution (manual adjust)	Maximum desired deceleration
Maximum recorded deceleration (Minimum)	Bmax_min	m/s^2^	Desired deceleration distribution (manual adjust)	Minimum desired deceleration
Positive accumulated acceleration per kilometre	PAA_km	m/s^2^ km	Calibration of car following model (headway)	Calibration of car following model (headway)

**Table 3.** Changes in vehicle trajectory variables produced by the activation of a section speed control system.

**Parameters**	**Base Scenario**	**Section Speed Control Activated**	**Variation %**
VP95_mean ***	90.2	88.8	−1.6%
VP95_Sd	3.9	3.0	−23.1%
VP95_max	101.2	98.4	−2.8%
VP95_min	78.9	79.9	1.3%
Amax_mean ^†^	0.8	0.8	0.0%
Amax_Sd	0.2	0.3	50.0%
Amax_max	1.6	2.3	43.8%
Amax_min	0.3	0.4	33.3%
Bmax_mean ***	0.9	1.0	11.1%
Bmax_Sd	0.4	0.3	−25.0%
Bmax_max	2.4	2.1	−12.5%
Bmax_min	0.4	0.4	0.0%
PAA_km ^†^	3.4	3.3	−2.9%
Fuel consump. ***	4.67	4.49	−3.8%

* Significant at *p* < 0.1; ** Significant at *p* < 0.05; *** Significant at *p* < 0.01; † No statistically significant differences between group means.

**Table 4.** Changes in vehicle trajectory variables produced by the activation of a Variable Speed Limit system.

**Parameters**	**Base Scenario**	**Variable Speed Limits Activated**	**Reduction %**
VP95_mean ^†^	90.9	90.9	0.0%
VP95_Sd	4.2	3.2	−23.8%
VP95_max	101.3	96.9	−4.3%
VP95_min	76.2	85.8	12.6%
Amax_mean **	0.9	1.2	33.3%
Amax_Sd	0.6	0.6	0.0%
Amax_max	3.0	3.1	3.3%
Amax_min	0.3	0.5	66.7%
Bmax_mean *	1.3	1.5	15.4%
Bmax_Sd	0.9	0.7	−22.2%
Bmax_max	3.4	3.0	−11.8%
Bmax_min	0.3	0.4	33.3%
PAA_km ^†^	5.1	6.2	21.6%
Fuel consump. ^†^	4.13	4.05	−1.94%

* Significant at *p* < 0.1; ** Significant at *p* < 0.05; *** Significant at *p* < 0.01; **^†^** No statistically significant differences between group means.

**Table 5.** Changes in vehicle trajectory variables produced by the use of a Cruise Control system.

**Parameters**	**Base Scenario**	**Cruise Control Activated**	**Variation %**
VP95_mean **	92.5	91.0	−1.6%
VP95_Sd	1.4	1.6	14.3%
VP95_max	95.1	94.8	−0.3%
VP95_min	90.3	88.4	−2.1%
Amax_mean *	0.9	1.0	11.1%
Amax_Sd	0.3	0.2	−33.3%
Amax_max	1.4	1.3	−7.1%
Amax_min	0.6	0.8	33.3%
Bmax_mean ^†^	0.9	0.8	−11.1%
Bmax_Sd	0.4	0.2	−50.0%
Bmax_max	1.9	1.4	−26.3%
Bmax_min	0.5	0.6	20.0%
PAA_km ***	3.4	1.8	−47.1%
Fuel consump. ^†^	3.61	3.44	−4.70%

* Significant at *p* < 0.1; ** Significant at *p* < 0.05; *** Significant at *p* < 0.01; **^†^** No statistically significant differences between group means.

**Table 6.** Changes in vehicle trajectory variables produced by eco-driving behaviour in motorway itineraries.

**Parameters**	**Base Scenario**	**Eco-Driving Motorway**	**Variation %**
VP95_mean ^†^	91.9	91.6	−0.3%
VP95_Sd	4.0	2.8	−30.0%
VP95_max	98.9	97.9	−1.0%
VP95_min	71.7	83.7	16.7%
Amax_mean ***	0.9	0.8	−11.1%
Amax_Sd	0.3	0.3	0.0%
Amax_max	2.0	1.8	−10.0%
Amax_min	0.4	0.3	−25.0%
Bmax_mean ***	1.0	0.7	−30.0%
Bmax_Sd	0.4	0.5	25.0%
Bmax_max	2.4	2.9	20.8%
Bmax_min	0.4	0.3	−25.0%
PAA_km ***	4.6	3.8	−17.4%
Fuel consump. **	4.18	3.82	−8.61%

* Significant at *p* < 0.1; ** Significant at *p* < 0.05; *** Significant at *p* < 0.01; **^†^** No statistically significant differences between group means.

**Table 7.** Changes in vehicle trajectory variables produced by eco-driving behaviour in urban itineraries.

**Parameters**	**Base Scenario**	**Eco-Driving Urban**	**Variation %**
VP95_mean ***	53.5	50.1	−6.4%
VP95_Sd	6.1	6.0	−1.6%
VP95_max	79.0	65.8	−16.7%
VP95_min	34.1	37.5	10.0%
Amax_mean ***	2.0	1.8	−10.0%
Amax_Sd	0.3	0.3	0.0%
Amax_max	2.7	2.6	−3.7%
Amax_min	1.1	0.7	−36.4%
Bmax_mean ***	2.3	2.1	−8.7%
Bmax_Sd	0.6	0.6	0.0%
Bmax_max	5.3	5.5	3.8%
Bmax_min	1.2	1.1	−8.3%
PAA_km ***	36.9	32.6	−11.7%
Fuel consump. ***	4.34	4.03	−7.14%

* Significant at *p* < 0.1; ** Significant at *p* < 0.05; *** Significant at *p* < 0.01; † No statistically significant differences between group means.
